# A secure visualization platform for pathogenic genome analysis with an accurate reference database

**DOI:** 10.1016/j.bsheal.2024.07.003

**Published:** 2024-07-10

**Authors:** Guomei Fan, Chongye Guo, Qian Zhang, Dongmei Liu, Qinglan Sun, Zhigang Cui, Haijian Zhou, Yuanchun Zhou, Zhibin Guo, Juncai Ma, Linhuan Wu

**Affiliations:** a Microbial Resource and Big Data Center, Institute of Microbiology, Chinese Academy of Sciences, Beijing 100101, China; b Chinese National Microbiology Data Center (NMDC), Beijing 100101, China; c National Key Laboratory of Intelligent Tracking and Forecasting for Infectious Diseases, National Institute for Communicable Disease Control and Prevention, Chinese Center for Disease Control and Prevention, Beijing 102206, China; d Computer Network Information Center, Chinese Academy of Sciences, Beijing 100190, China; e State Key Laboratory of Microbial Resources, Institute of Microbiology, Chinese Academy of Sciences, Beijing 100101, China

**Keywords:** Pathogen surveillance, One-stop analysis tools, Sequence typing, Horizontal transfer, Secure computing

## Abstract

Investigating the genetic and developmental characteristics, infection transmission attributes, and epidemiological trends of pathogens using genomic data represents the foundation for pathogen surveillance and is a crucial prerequisite for guaranteeing global health security. To meet the analytical demands of research relating to pathogen prevention and control, we designed a secure visualization system capable of pathogen genome assembly, annotation, species identification, sequence typing, antibiotic resistance and virulence analysis, genomic mobile element and transferable resistance gene annotation, and phylogenetic tree reconstruction. For highly pathogenic organisms requiring complete data protection, we have developed a secure computing tool that utilizes a trusted execution environment, is combined with blockchain and privacy computing technologies, and is specifically designed for nucleotide basic local alignment search tool (BLASTn) comparison analysis. This technological advancement offers scientific support for in-depth investigations into pathogen transmission and epidemiological mechanisms, environmental adaptability, evolutionary trends, and immune evasion mechanisms, as well as the identification of new or emerging pathogen strains. This, in turn, aids efforts in infectious disease prevention, treatment, and research.

## Highlights

• **Scientific question**: There is a lack of a system specifically designed for the comprehensive analysis of pathogenic microbial genomes. Existing tools, while diverse, lack unity and standardization, and their species coverage is relatively limited in terms of diversity and breadth.
• **Evidence before this study:** Although the Bacterial and Viral Bioinformatics Resource Center (BV-BRC provides a wealth of bacterial, archaeal, and viral resources, the analysis tools are not sufficiently systematic. They mostly focus on genome comparison and genetic phylogenetic tree drawing, without functional gene annotation and analysis. Enterobase and the Public Databases for Molecular Typing and Microbial Genome Diversity (PubMLST), as the main reference databases for pathogen typing research, only provide typing results for a small amount of pathogenic bacteria. Specifically, Enterobase only publishes typing results for nine species.
• **New findings**: Our study established a one-stop analysis system that enables genome assembly, annotation, species identification, sequence typing with in-house schemas for 112 species, antibiotic resistance and virulence annotation, genomic mobile element and transferable resistance gene annotation, as well as the construction of phylogenetic trees. At the same time, we have also developed in-house schemas for species lacking public options, to ensure consistent and efficient analysis.
• **Significance of the study:** The study developed a secure analysis tool for highly pathogenic organisms, utilizing trusted execution environment, blockchain, and privacy computing specifically for nucleotide basic local alignment search tool (BLASTn) comparison. This, marks the first secure platform for complete data protection in this field.

## 1 Introduction

The surveillance of pathogens of public health importance, routinely conducted in communities or the environment, involves the monitoring of both known and emerging pathogens, thus providing insights into the emergence and spread of infectious diseases [1]. Genomics-based pathogen surveillance plays a crucial role in the efficient detection of infectious diseases, facilitating high-resolution source tracing, and informing decisions for disease control measures [2]. Furthermore, pathogen genomics has proven successful in assessing drug susceptibility in individual patients [3] and has also played a vital role in understanding pathogen immune escape mechanisms during immunotherapy and vaccine development [4], [5].

Recently, the World Health Organization has emphasized the prioritizing of pathogen genomic surveillance within public health systems [6]. Achieving accurate surveillance involves understanding the genetic and developmental characteristics, infection transmission attributes, and spatiotemporal epidemiological trends of pathogens, which requires the integration of genomics and epidemiological data [7], [8], [9]. Developing analytical tools and highly accurate reference databases is fundamental for supporting pathogen genome surveillance. Accordingly, there is a need for more accurate, efficient, and systematic genomics analysis tools, along with strategies that reduce the time and labor costs associated with related research.

## 2 Materials and methods

To address the analytical needs of pathogen prevention, control, and research programs, we have designed a one-stop analytical system specifically tailored for pathogen genomics research. This system is built upon the Global Catalogue of Pathogens (gcPathogen), a high-quality reference database with comprehensive pathogen coverage established in our previous work [10]. The software package and privacy computing program are freely accessible without the need for registration through gcPathogen at https://nmdc.cn/gcpathogen/tools. Although these tools were briefly mentioned at the time, this study provides a more detailed introduction to the specific usage of each tool and the reference database they rely on, as they play a crucial role in the data analysis, as presented here.

**Fig. 1:**
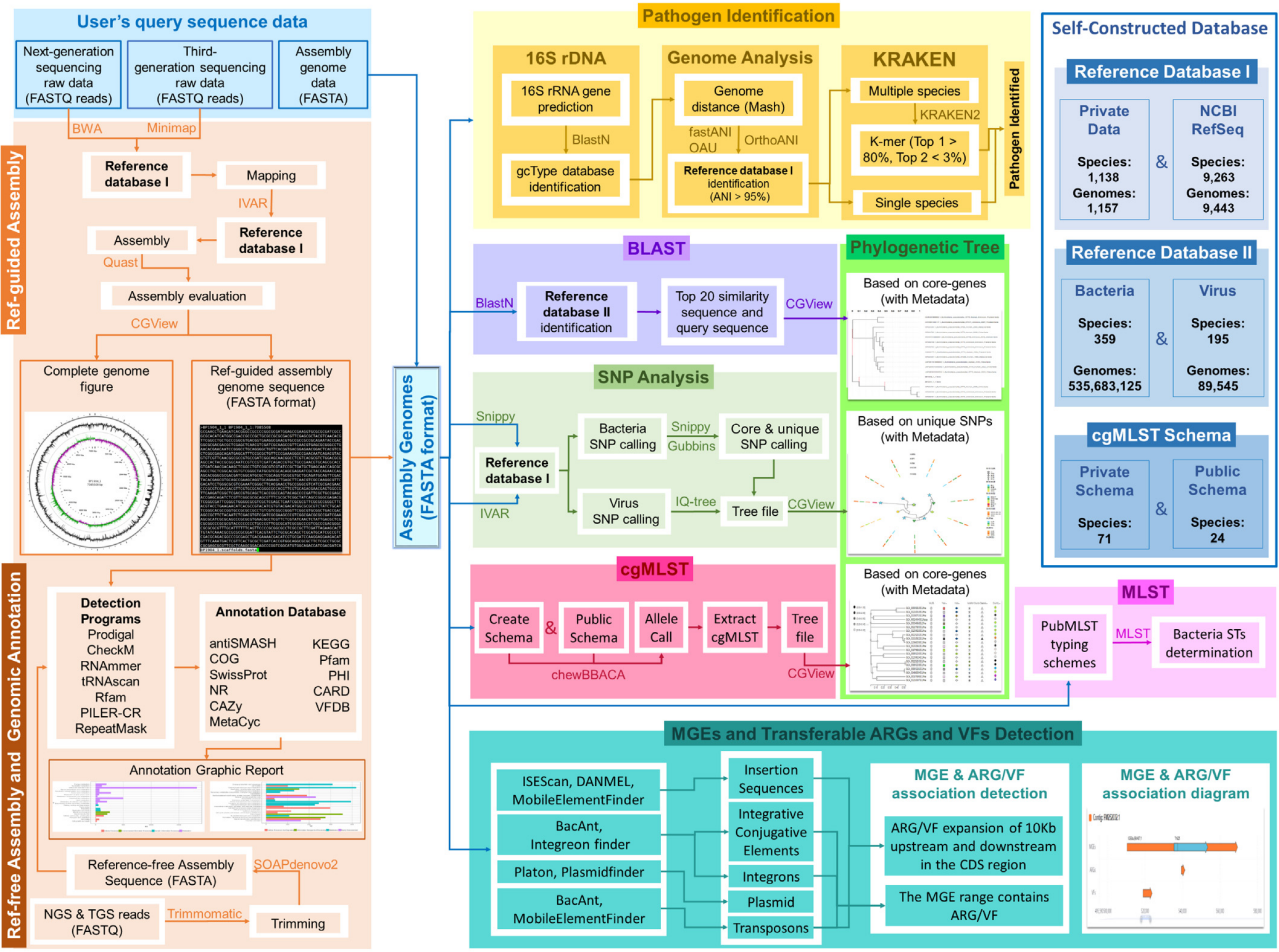
Processing workflow of the Global Catalogue of Pathogens (gcPathogen) one-stop analysis system. Abbreviations: MLST, multilocus sequence typing; cgMLST, core genome multilocus sequence typing; SNP, single nucleotide polymorphism; MGEs, mobile genetic elements; ARGs, transferable antibiotic resistance genes; VFs, virulence factors; DNA, deoxyribo nucleic acid; BLAST, basic local alignment search tool; NGS, next-generation sequencing; TGS, third-generation sequencing; NCBI, National Center for Biotechnology Information.

**Fig. 2:**
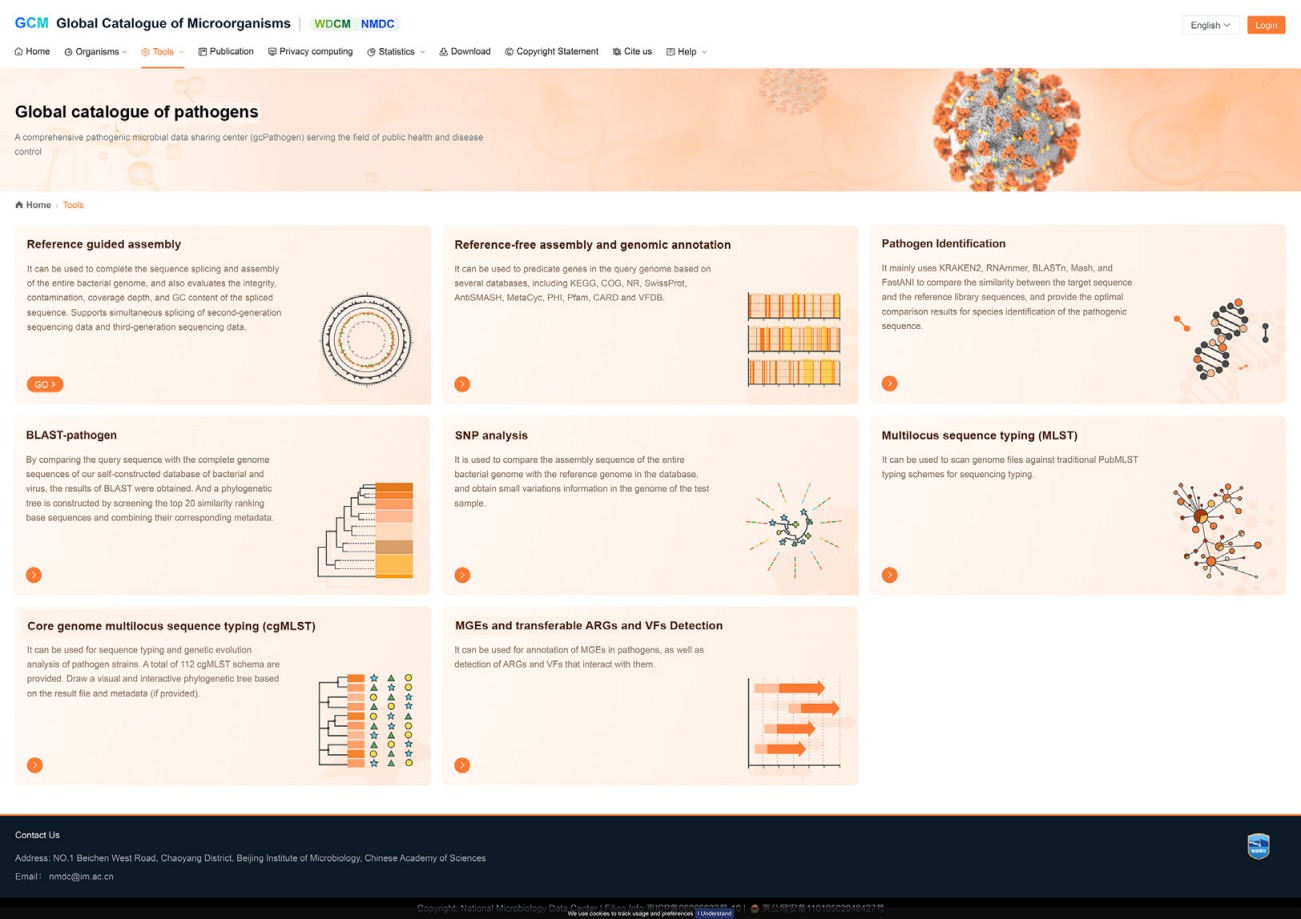
The webpages for online data analysis pipelines. Abbreviations: BLAST, basic local alignment search tool; SNP, single nucleotide polymorphism; MLST, multilocus sequence typing; cgMLST, core genome multilocus sequence typing; MGEs, mobile genetic elements; ARGs, antibiotic resistance genes; VFs, virulence factors.

Our system facilitates standardized assembly, pathogen species identification, and similarity search [basic local alignment search tool (BLAST)-pathogen]. In addition, the system supports pathogen typing [multilocus sequence typing (MLST), core genome multilocus sequence typing (cgMLST), and single nucleotide polymorphism (SNP) analysis], phylogenetic tree reconstruction, genomic annotation, and the detection of mobile genetic elements (MGEs), transferable antibiotic resistance genes (ARGs), and virulence factors (VFs) (Figs. 1 and 2). These functionalities provide comprehensive support for in-depth studies on pathogen transmission and epidemiological mechanisms, the determination of environmental adaptability and evolutionary trends, the analysis of pathogen immune escape mechanisms, and the identification of new or emergent subtypes. The specific processes, analysis parameters, and detailed information on software usage for all of the following tools are listed in Supplementary material.

### 2.1 Genomic assembly

Faced with the challenge of massive dataset assembly, two methods are commonly used, namely, assembly with and assembly without a reference sequence. The former approach can achieve rapid assembly, thus meeting the requirements of time-sensitive tasks. In contrast, the latter approach, which primarily relies on the overlapping regions of the original reads for assembly, is not limited by the complexity of species genome and type, making it more suitable for assembling the genomes of new species [11].

#### 2.1.1 Reference-guided assembly

Initially, the reference-guided assembly tool employs the National center for biotechnology information (NCBI) RefSeq collection [12], which contains reference genomes for 12,364 species of bacteria, totalling 12,750 genomes. To enhance its utility, a reference database for validly published species was established, incorporating 2,796 genomes from 2,774 species that were assembled by the gcType 10 K sequencing project [13]. This reference library aims to meet the assembly requirements of a wide range of known bacterial species, thus offering a more precise reference (Supplementary material S1).

#### 2.1.2 Reference-free assembly

The detection of new or unknown pathogens in disease control efforts may necessitate the *de novo* assembly of sequencing data. In this pipeline, raw reads (FASTQ format) derived from next-generation sequencing (NGS) and third-generation sequencing (TGS) undergo quality control using Trimmomatic [14]. Subsequently, based on different data types and assembly requirements, the appropriate software is automatically chosen to assemble these sequences, generating a genome sequence in FASTA format (Supplementary material S2).

### 2.2 Pathogen identification and basic local alignment search

#### 2.2.1 Pathogen identification

16S rDNA sequences, consisting of highly conserved and variable regions, are ubiquitous in prokaryotes. Researchers utilize the discriminatory potential of these sequences to determine phylogenetic relationships among bacterial genera [15]. However, 16S rDNA sequencing has limitations in resolving bacterial strains at the species level. To achieve species-level identification, the average nucleotide identity (ANI) is used to determine the average nucleotide similarity between homologous sequences derived from whole-genome sequencing [16]. However, certain pathogenic strains may display close genetic relationships, and thus high nucleotide similarity, thereby posing challenges for discrimination. For instance, strains of *Escherichia coli* (*E. coli*) and *Shigella* within the Enterobacterales are difficult to distinguish through ANI methods [17]. To address these challenges and allow for accurate strain identification, more precise comparison methods are necessary. We have developed a comprehensive set of tools for accurate pathogen species identification. In summary, the tool first employs RNAmmer and BLASTn for the initial identification of the 16S rDNA gene. Subsequently, the tool utilizes the USEARCH algorithm within OAU to perform a local alignment and calculates the ANI value between the two genomes. Following this, OrthoANI is used to calculate the ANI between the genomes, which involves a sequence comparison methodology distinct from the BLAST algorithm. Finally, species-level identification is determined through the FastANI algorithm by comparing the query genome against the gcPathogen Pan unique core-gene reference library to calculate ANI values and establish the closest species match. The versions of each software and the parameters that are used are listed in Supplementary material S3.

#### 2.2.2 BLAST-pathogen

Various public databases associated with pathogenic microorganisms employ NCBI-BLAST tools for rapid sequence alignment. The alignment reference database, NCBI-nr, contains screened non-redundant protein sequence information from diverse species, thus minimizing redundancy in sequence data. As an alternative, a dedicated reference database for pathogenic bacteria and viruses was established, comprising 465,736 high-quality genomes from 359 human pathogenic bacteria and 53,568,325 high-quality contigs. This database also incorporates 7,787 high-quality genomes and 1,101,148 high-quality contigs from 195 viruses (Supplementary material S4-Table S3). Each genome is connected to at least one piece of additional information, known as metadata (Supplementary material S4). Search results can further be refined to create a phylogenetic tree, which depicts the evolutionary relationships between organisms. This tool not only expedites the comparison of pathogen sequences with increased accuracy and informativeness but also allows users to explore the evolutionary relationships of sequences, aiding in understanding their routes of transmission and sources of isolation.

### 2.3 Pathogen sequence typing

Pathogen typing studies play a crucial role in unveiling genetic differences and relationships among pathogenic strains, as well as aiding in source tracing. Various typing methods offer different levels of resolution. MLST involves aligning the sequences of housekeeping genes, providing a straightforward approach for establishing genetic relationships among strains. It assigns strains of the same serotype to distinct sequence types (STs). However, it has limited resolution, which hinders its effectiveness in typing closely related strains. cgMLST operates on the core genome, utilizing core genes identified from a large number of strains as sequence typing markers. This method excels in high-resolution species typing, contributing to the study of strain population structures, genetic evolution, and rapid source tracking [18]. SNP typing is a genome typing method centered on single base variations in the genome. SNP analysis offers a higher resolution than cgMLST because it additionally considers intergenic regions. Consequently, it is well-suited for etiological tracing analysis and finds widespread use in the etiological classification, traceability, and prevention of pathogenic bacteria [18], [19]. We have established a web-based MLST tool [20] to facilitate the comparison of sequences of seven housekeeping genes, which assigns strains of the same bacterial genus to distinct STs. Moreover, the cgMLST schema of 112 species established by the gcPathogen platform is established. The SNP analysis pipeline is based on the construction of a core SNP matrix (Supplementary material S5).

### 2.4 Phylogenetic tree construction

The creation of a phylogenetic tree for pathogens, coupled with epidemiological metadata, is instrumental in comprehending the genetic traits and evolution paths of pathogens, as well as aiding in the tracking of transmission sources [21]. We have devised a pipeline in which cgMLST or SNP analysis is employed to generate the tree file, which is then visualized using CGView [22]. Users have the option of uploading associated metadata for sequences, encompassing details such as ST, sampling location, sampling time, host, host disease, source of isolation, and other pertinent information. These metadata are annotated on the respective sequences, facilitating comparison and providing insights for epidemiologic traceability.

### 2.5 Genomic annotation

The annotation and prediction of genes in pathogen genomes are pivotal for the understanding of gene function, for the identification of potential drug targets, and for informing the development of disease diagnosis, treatment, and epidemic prevention programs.

**Fig. 3:**
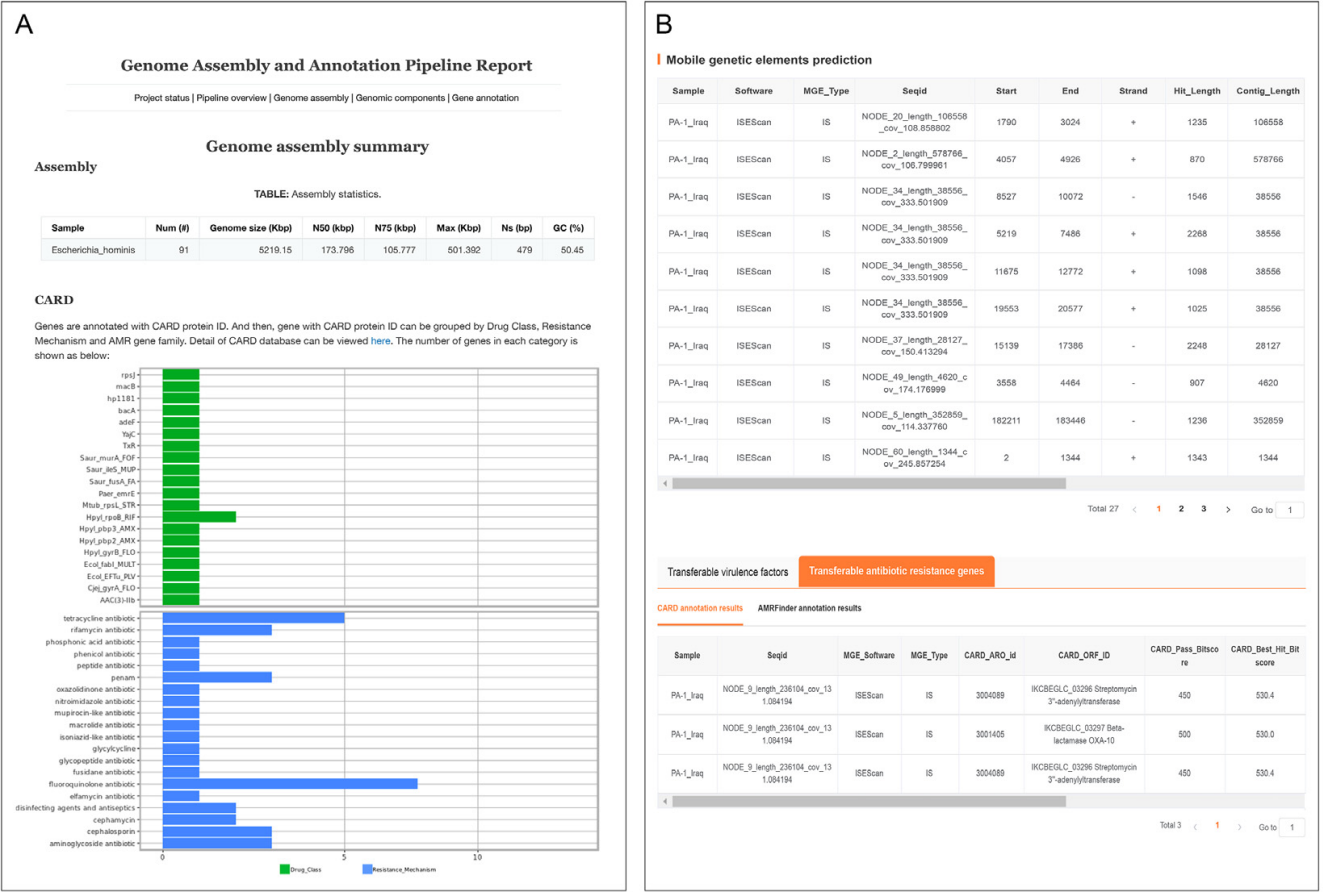
Results display pages for the genome assembly and annotation pipeline (A) and mobile genetic element (MGE) analysis (B). Abbreviation: CARD, Comprehensive Antibiotic Resistance Database.

The online tools that are widely used for genome functional gene annotation are often tailored to a single database. We have developed an all-in-one annotation tool capable of annotating protein functions, biological pathways, gene product interactions, secondary metabolites, ARGs [Comprehensive Antibiotic Resistance database (CARD)] [23], VFs [Virulence factors of Pathogenic Bacteria database (VFDB)] [24], and pathogen-host relationships (Supplementary material S6). The results are displayed on a dedicated page, as shown in Fig. 3A.

### 2.6 MGE, ARG, and VF detection

The study of ARGs enables an in-depth understanding of the adaptability of pathogens to drugs and the evolution of drug resistance among pathogens, offering scientific guidance for the development of new antibacterial drugs and rational drug use. Additionally, the study of VFs allows researchers to focus on key molecules related to pathogen invasion, reproduction, and immune escape. MGEs can carry and transfer ARGs and VFs, thus promoting the evolution and adaptation of pathogens [25].

There is no standardized and unified annotation process for the detection of MGEs, ARGs, and VFs. Therefore, we have developed an integrated pipeline that can detect insertion sequences, integrative and conjugative elements (ICEs), integrons (INs), plasmids, phages, and transposons (Tn), providing synchronous alignment and annotation. Subsequently, Diamond [26] is used to predict ARGs by aligning sequences against the CARD database, and VFs by aligning sequences against the VFDB database. The criteria for determining the horizontal transferability of ARGs and VFs are listed in Supplementary material S7 and the results display page is depicted in Fig. 3B.

### 2.7 Secure computing based on privacy computing and blockchain

Genomic data of pathogens hold immense value in clinical diagnosis, disease control, and drug development. However, due to security concerns, sensitive pathogenomic data cannot be fully open for transfer and computation before formal release. To address this issue, we have designed and implemented a sequence similarity alignment platform utilizing blockchain and privacy computing to transfer and compute this data securely.

**Fig. 4:**
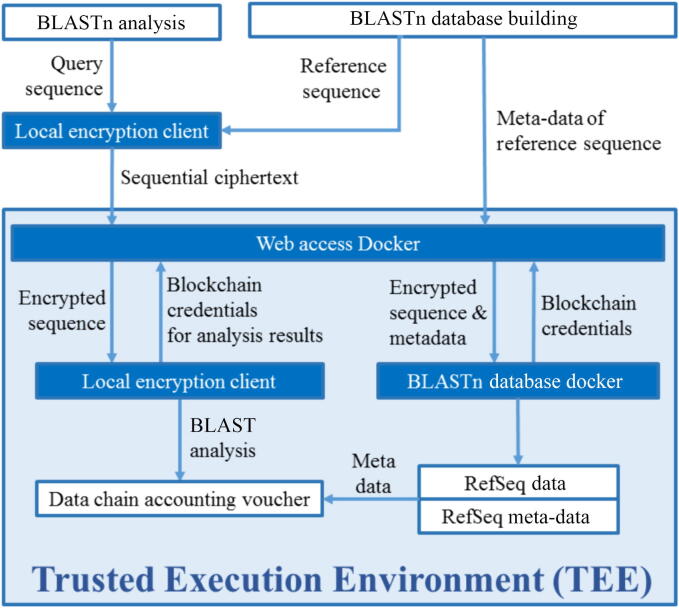
The processing workflow of secure computing. Abbreviation: BLASTn, nucleotide basic local alignment search tool.

Blockchain, known for its security and traceability through the hierarchical registration of transaction lists, plays a crucial role in the security of the data transfer process, functioning like a tamper-proof electronic public ledger that permanently records the history of all transactions. Its key feature, “decentralization”, means that no central authority or server controls this notebook. Every participant has a complete copy and can mutually verify it, thus ensuring data security and credibility. A trusted execution environment (TEE), providing a protected and isolated memory area, ensures data confidentiality (Fig. 4). The TEE is an isolated environment provided within the host processor or device, which can protect the applications running in it from interference by other applications or malware in the operating system, ensuring the security and processing of data. In this pipeline, users submit their encrypted genomic sequences to the server equipped with a TEE. The BLASTn reference database is integrated into the TEE, allowing data analysis to be performed securely within this environment. Once the analysis is completed within the TEE, the results are securely sent back to the submitter. All intermediate or temporary plaintext data generated during the processing is immediately and securely destroyed. Every step of this process is recorded by the blockchain, ensuring transparency and traceability throughout the entire procedure. By utilizing cryptography, privacy computing, and blockchain technology, we can guarantee that all parties involved, including analysis service providers, cannot access the plaintext content of the data, significantly enhancing the security of privacy computing.

## 3 Results

### 3.1 Case comparison between the gcPathogen one-stop analysis system and analysis tools from other platform

To illustrate the differences between the various tools in the gcPathogen one-stop analysis system and related tools on other platforms, a case study was conducted using public data from *Shigella flexneri* in NCBI. We compared the tools in the gcPathogen analysis system with those in other analysis platforms in terms of functionality and usage conditions (Table 1).

**Table 1:** Comparison of website tools for analyzing various pathogenic microorganisms.

Website tools	Species identification	Basic local alignment search tool (BLAST) with phylogenetic tree	Genomics analysis service (mapping/alignment/annotation)	Multilocus sequence typing (MLST)	Core genome multilocus sequence typing (cgMLST)	Single nucleotide polymorphism (SNP)	Antibiotic resistance genes (ARG) annotation	Virulence factors (VF) annotation	Mobile genetic elements (MGE) annotation	Antibiotic resistance genes and virulence factors associated with mobile genetic elements annotation	Website address
The Global Catalogue of Pathogens (gcPathogen)-one-stop-analysis system	✔	✔	✔	✔	✔	✔	✔	✔	✔	✔	https://nmdc.cn/gcpathogen/
National center for biotechnology information (NCBI)-Pathogen Detection							✔	✔			https://www.ncbi.nlm.nih.gov/pathogens/
Bacterial and viral bioinformatics resource center (BV-BRC)			✔			✔	✔	✔			https://www.bv-brc.org/
EnteroBase				✔	✔						https://enterobase.warwick.ac.uk/
The Eukaryotic Pathogen, Vector and Host Informatics Resources (VEuPathDB)			✔	✔							https://veupathdb.org
Pathogenwatch				✔	✔		✔				https://pathogen.watch/
Public databases for molecular typing and microbial genome diversity (PubMLST)				✔	✔						https://pubmlst.org/
The type (strain) genome server (TYGS)	✔										https://tygs.dsmz.de/
proMGE									✔		https://promge.embl.de/
VRprofile2									✔	✔	https://tool2-mml.sjtu.edu.cn/VRprofile/home.php

### 3.2 Comparison of pathogen identification between gcPathogen and the type (strain) genome server (TYGS)

**Fig. 5:**
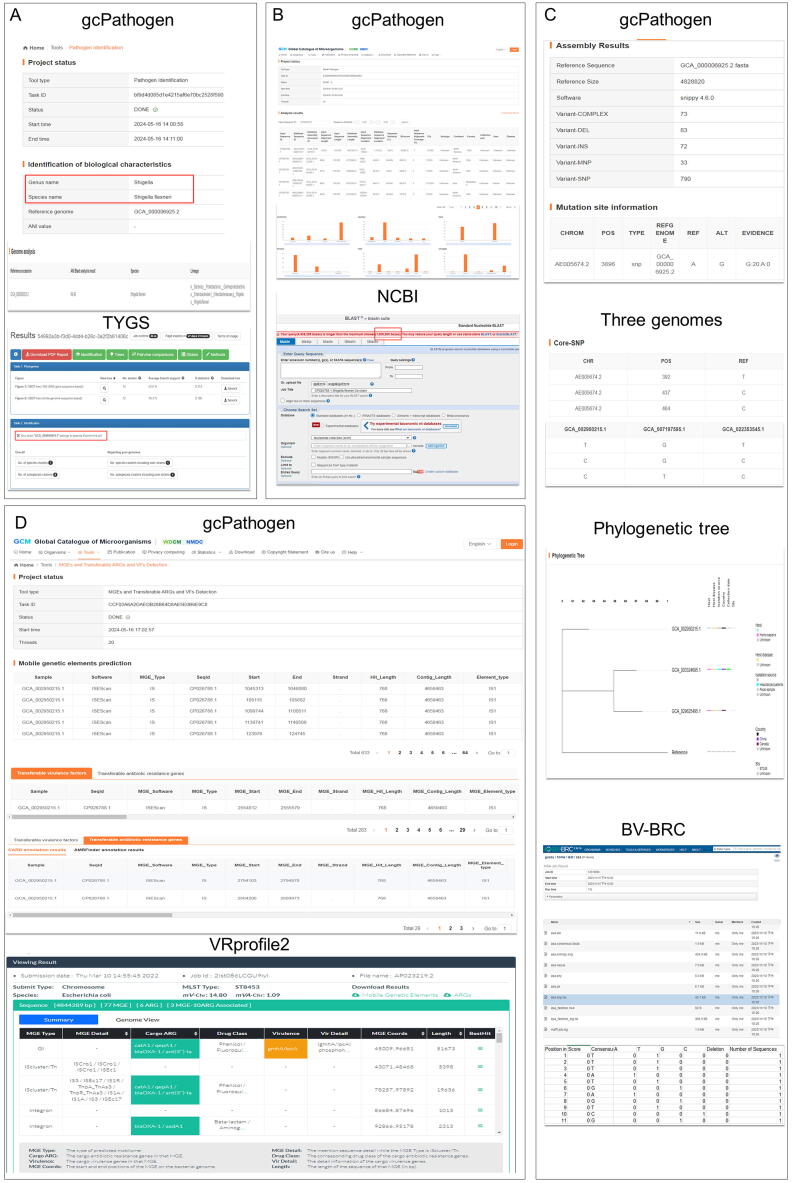
The comparison of the Global Catalogue of Pathogens (gcPathogen) one-stop analysis system tool with tools from other platforms. A) Comparison of pathogen identification between gcPathogen and the type (strain) genome server (TYGS). B) The basic local alignment search tool (BLAST)-pathogen identification in gcPathogen *vs.* nucleotide basic local alignment search tool (BLASTn) in National Center for Biotechnology Information (NCBI). C) Comparison of single nucleotide polymorphism (SNP) analysis between gcPathogen and bacterialand viral bioinformatics resource center (BV-BRC). D) Comparison of mobile genetic element (MGE) and transferable antibiotic resistance gene (ARG), and virulence factor (VF) detection between gcPathogen and VRprofile2.

We used the species identification tool in TYGS [27] and the pathogen identification tool in gcPathogen to identify genome GCA_002950215.1 of *Shigella flexneri*. The result of the analysis using TYGS identified the species of this genome as *E*. *coli*. However, in gcPathogen, the genome was identified as *Shigella flexneri* (Fig. 5A). This suggests that different identification tools may yield conflicting results, highlighting the importance of considering multiple lines of evidence and using a range of tools to ensure the reliability of identification, especially when dealing with closely related organisms or those with complex taxonomic histories.

### 3.3 BLAST-pathogen versus NCBI-BLASTn

Using genome GCA_002950215.1 downloaded from NCBI as the test data, after uploading it to the BLAST-Pathogen tool, 300 alignment results were obtained, all of which were *Shigella flexneri*. Among them, there were 35 sequences with a similarity of 100 %. As shown in Fig. 5B, in addition to providing basic information such as sequence length, base position, and similarity, the analysis also yielded the corresponding species, serotype, ST, sample collection location, sample collection time, host, and host disease information for the aligned sequences. Simultaneously, the relevant statistical information of these sequences was visually displayed. Conversely, given that the uploaded sequence length was greater than 1,000,000 bp, the analysis could not be performed using NCBI-BLASTn [28].

### 3.4 Comparison of SNP analysis between gcPathogen and bacterial and viral bioinformatics resource center (BV-BRC)

When using the SNP analysis tool of gcPathogen, there will be some differences in the analysis results depending on whether one genome or more than three genomes are uploaded. When only uploading genome GCA_002950215.1, a total of 1,050 mutations were aligned, including 83 deletions, 72 insertions, and 790 SNPs. The results file displays the locations and reference serial numbers of these SNPs, as well as variation site information. If more than three genomes (GCA_002950215.1, GCA_003324695.1, and GCA_029625495.1) are uploaded simultaneously along with their corresponding metadata, specific information about the core SNPs of these three genomes will be obtained, including variant site locations, reference genomes, and mutation status at variant sites for each genome. Additionally, a phylogenetic tree associated with metadata will be generated to illustrate the genetic distance between these genomes (Fig. 5C). The results of the multiple sequence alignment and SNP / variation analysis tool in BV-BRC [29] mainly consist of a list of mutation sites, without a phylogenetic tree.

### 3.5 Comparison of the detection of MGEs and transferable ARGs and VFs between gcPathogen and VRprofile2

The MGE and transferable ARG and VF detection tool in gcPathogen supports the analysis of MGEs and their associated ARGs and VFs in genomic data with multiple sequences. As shown in Fig. 5D, when using this tool in gcPathogen to analyze multiple-sequence genomic data (GCA_002950215.1), the results file listed a total of 633 MGE sequences from 19 MGE families. Among the MGEs, IS1 and IS3 were associated with 29 ARGs, while IS1, IS3, IS4, IS110, IS91, and the plasmid IncFII were related to 283 VFs. On the other hand, VRprofile2 [30] can only annotate MGEs and their associated ARGs and VFs in a single sequence, and thus cannot be used when genomes containing multiple sequences are uploaded.

## 4 Discussions and conclusion

After comparing tools from different platforms, we found that although genome assembly and annotation are widely used techniques, many current online analysis platforms separate these two processes into distinct workflows. However, our tool integrates these two processes and divides the online interface into four sections tailored to the potential needs of users − assembly, assembly and annotation, genome annotation, and gene annotation. This flexibility allows users to choose the options that best meet their analytical requirements.

For species identification tools, the comprehensiveness of the reference database is crucial. Our species identification reference database comprises not only 12,750 reference sequences from 12,364 bacterial species in NCBI RefSeq, but also encompasses 2,796 self-tested reference genomes representing 2,774 distinct species. This enables our tool to identify a broader range of species.

Compared to other platforms, the primary advantage of the BLAST-Pathogen tool is that it can furnish users with statistical results pertaining to continents, countries, hosts, diseases, MLSTs, and serotypes while aligning sequences based on a public database. Moreover, in NCBI’s BLASTn tool, users cannot directly upload complete genome assembly data for alignment due to a limitation of sequence length, which cannot exceed 1,000,000 bases. Conversely, BLAST-Pathogen allows users to directly upload complete genome sequences for alignment. Additionally, BLAST-Pathogen employs a self-constructed library that offers more accurate and representative genomes as references, thereby avoiding a large amount of redundant and interfering data.

The main limitation of the cgMLST tool is in the extraction of allelic schema. Currently, there are three primary online tools capable of performing cgMLST—EnteroBase, which offers a total of nine schemas; public databases for molecular typing and microbial genome diversity (PubMLST), which offers 16 schemas; and PathogenWatch, presenting 16 schemas. However, the gcPathogen-cgMLST tool grants access to and enables the downloading of schemas for 112 pathogenic bacterial species, thereby satisfying the needs of cgMLST for a broader range of species.

Compared to similar tools on other platforms, the foremost advantage of our SNP analysis tool is that it not only generates SNP analysis results but also presents phylogenetic tree results in graphical form. Furthermore, it enables users to upload metadata information corresponding to each sequence, including host, host disease, isolation source, country, collection date, and ST. This metadata information is further displayed on the phylogenetic tree, offering users a more intuitive visualization of the analysis results.

Furthermore, existing analysis platforms do not offer comprehensive annotation for MGEs or transferable ARGs and VFs. Only VRprofile2 can simultaneously annotate MGEs and transferable ARGs and VFs. However, unlike VRprofile2, which can only handle the upload of one sequence at a time, the gcPathogen tool supports the simultaneous analysis of multiple sequences.

In conclusion, gcPathogen, a comprehensive one-stop analysis system tailored for pathogen prevention, control, diagnosis, and research, excels in swiftly and accurately detecting and identifying human pathogenic bacteria and viruses. gcPathogen facilitates rapid and high-precision genome assembly and supports various analyses such as MLST, cgMLST, SNP typing, and phylogenetic tree construction for epidemiological transmission traceability as well as transmission route and transmission trend analysis. Additionally, the system enables the *de novo* assembly of new or unknown pathogenic bacteria and the annotation of multiple functional genes. The system further supports the analysis of the horizontal transfer of ARGs and VFs, offering valuable scientific guidance for clinical medication and epidemic prevention and control. Looking ahead, our goal is to expand and integrate additional functional analysis processes encompassing metagenomic analysis, drug resistance risk warning, mobile component analysis, and transfer risk warning. This ongoing development aims to provide even more comprehensive and accurate services for the prevention and treatment of infectious diseases.
