# Supplementary material for: A secure visualization platform for pathogenic genome analysis with an accurate reference database
Source: Biosaf Health. 2024 Jul 10;6(4):235–43. doi: 10.1016/j.bsheal.2024.07.003 (PMC11894998; doi:10.1016/j.bsheal.2024.07.003)
Supplement: Supplementary Data 1 [file mmc1.docx]

**Supplementary Material**

This section describes the tool flow and analysis parameters and provides detailed information relating to software usage in the gcPathogen one-stop analysis system.

**S1. Reference-guided Assembly**

The Reference-guided Assembly tool accommodates raw read data (in FASTQ format) from long-read platforms (TGS) such as PacBio and Oxford Nanopore, as well as short-read platforms (NGS), including Illumina and Ion Torrent. For NGS, BWA 0.7.17-r1188 [1] is employed to compare reads with the Reference Database, while Minimap 2: 2.24-r1122 is utilized for TGS [2]. Subsequently, iVar 1.3.1 [3] aligns the assembled sequences with Reference Database I. The completeness of the assembled genome is then assessed using Quast 5.0.2 [4], with the evaluated genome representing a complete high-quality genome. Finally, CGView [5], is employed to generate the genome map, which is simultaneously presented to the user along with FASTA file of the assembly genome.

**S2. Reference-free Assembly**

The assembly software employed includes 1) SOAPdenovo2, commonly used in bacterial and fungal genome assembly [6]; 2) SPAdes, which is suitable for bacterial and fungal genome assembly, and is capable of splicing Pacbio and Nanopore sequencing data from single-molecule platforms [7]; 3) Platanus_B, which is based on a k-mer counting algorithm for fast and efficient assembly [8]; 4) Velvet, which is based on the De Bruijn graph algorithm, and is suitable for high-coverage, short-read sequence assembly [9]; 5) IDBA-UD, which is based on an iterative graph algorithm, and is capable of identifying and assembling repetitive sequences and complex structures in the genome [10]; and 6) CheckM [11], which is used for evaluating the integrity and contamination rate of assembled sequences. An assembled result is considered a complete, high-quality genome when the integrity surpasses 95% and the contamination rate is less than 5%.

The insert length distribution is determined using Picard, which relies on the sorted BAM files generated by mapping reads to assembled contigs. These assembled contigs or scaffolds are then fragmented. The GC content and sequencing depth for each window are calculated using sliding windows with a size of 500 bp and a step size of 20 bp. The accompanying figure illustrates the data falling within the 97.5% confidence interval, excluding outliers. Additionally, when employing various assembly software packages, the k-mer parameters are adjusted according to reads length, as outlined in Table S1. All the arguments used for the assembly tools are listed in Table S2.

**Table S1. K-mer setting of assembly tools.**

| **Read length** | **SPAdes** | **IDBA-UD** | **Velvet** | **SOAPdenovo2** | **Platanus-b** |
| --- | --- | --- | --- | --- | --- |
| **50-70 bp** | -k 21,27,33,39,45;default | --mink 21 --maxk 45 --step 6;default | 45;default | -K 21 -m 45;default | -k 21 -K 0.3 -s 6;default |
| **70-100 bp** | -k 27,35,43,51,59;default | --mink 17 --maxk 59 --step 8;--mink 27 --maxk 67 --step 10;default | 67;default | -K 27 -m 67;default | -k 27 -K 0.4 -s 8;-k 17 -K 0.45 -s 10;default |
| **100-127 bp** | -k 21,29,37,45,53;-k 21,35,49,63,77,91;default | --mink 21 --maxk 53 --step 8;--mink 21 --maxk 91 --step 14;default | 91;default | -K 35 -m 91;default | -k 21 -K 0.35 -s 8;-k 21 -K 0.6 -s 14;default |
| **>127 bp** | -k 21,35,49,63,77;-k 17,39,61,83,105,127;default | --mink 21 --maxk 77 --step 14;--mink 17 --maxk 124 --step 22;default | 127;default | -K 39 -m 127;default | -k 21 -K 0.51 -s 14;-k 17 -K 0.82 -s 22;default |

**Table S2. Arguments of assembly tools.**

| **Tools or databases** | **Arguments and description** |
| --- | --- |
| **fastQC** | (Default) |
| **trimmomatic** | SLIDINGWINDOW:5:20 MINLEN:20 |
| **sickle** | (Default) |
| **SPAdes** | (several sets of kmers) --careful --sc --disable-gzip-output |
| **IDBA** | (several sets of kmers) --pre_correction |
| **Velvet** | (several sets of kmers) -cov_cutoff auto -exp_cov auto |
| **SOAPdenovo2** | (several sets of kmers) -R -d 1 -M 1 -D 1 -F |
| **Platanus-b** | (several sets of kmers) |
| **CANU** | (Default) |
| **flye** | (Default) |
| **Pilon** | (--changes --fix all) |
| **checkM** | (lineage_wf) |
| **PILER-CR** | (Default) |
| **tRNAScan-SE** | (-qQ -Y) |
| **RNAmmer** | (-multi) |
| **Prodigal** | (Default) |
| **TRF** | (2 7 7 80 10 50 500 -f -d -m -h) |
| **Diamond** | -e 1e-5 --id 40 --query-cover 40 --subject-cover 40 |
| **NR** | (Diamond) |
| **KEGG** | (Diamond) |
| **COG** | (Diamond) |
| **Pfam** | (pfam_scan.pl) |
| **TIGRfam** | (pfam_scan.pl) |
| **Rfam** | (Infernal) |
| **Swiss-Prot** | (Diamond) |
| **MetaCyc** | (Diamond) |
| **PHI** | (Diamond) |
| **CAZy** | (Diamond) |
| **Anti-SMASH** | (Diamond) |
| **CARD** | (Diamond) |
| **VFDB** | (Diamond) |

**S3. Pathogen Identification**

For pathogen identificatio, the first step is to use RNAmmer v.1.2 for predicting the 16S rDNA gene of the test data, followed by BLASTn against the 16S rDNA reference database [12]. The results (e-value ≤ 1e-5) are sorted based on the bit-score, and the top 10 results are retained. In the second step, ANI-based analysis is conducted using the Mash [13], OrthoANI v.1.40 [14], OAU v.1.2 [15], and fastANI 1.31 [16] tools. The consistency of the query genome with the reference genome is calculated and species with the closest distance and a consistency exceeding 95% are retained. If the hits belong to the same species, the identification results are reported. If there are multiple species in the list, the pipeline automatically initiates k-mer-based analysis through KRAKEN2 v.2.09-beta [17]. If the k-mer abundance of the first species is higher than 80% and the k-mer abundance of the second species is lower than 3%, the genome is considered pollution-free and species-specific. This information is then combined with the ANI results and, if both results indicate the same species, that species is reported. If the two results suggest different species, the species with the highest agreement is identified.

**S4. BLAST-Pathogen**

Each genome is linked to at least one piece of metadata information, encompassing sampling time, sampling country, host, host disease, isolation source, ST, and serotype. The hit results can be further used to construct a phylogenetic tree. Metadata information for each sequence is annotated on the phylogenetic tree. The pathogens in the reference database are listed in Table S3.

**Table S3. The pathogens in the BLAST-Pathogen reference database.**

| **Pathogen name** | **Superkingdom** | **Pathogen name** | **Superkingdom** |
| --- | --- | --- | --- |
| Achromobacter piechaudii | Bacteria | Sarcina ventriculi | Bacteria |
| Achromobacter ruhlandii | Bacteria | Serratia liquefaciens | Bacteria |
| Acinetobacter baumannii | Bacteria | Serratia marcescens | Bacteria |
| Acinetobacter bereziniae | Bacteria | Serratia plymuthica | Bacteria |
| Acinetobacter colistiniresistens | Bacteria | Serratia rubidaea | Bacteria |
| Acinetobacter courvalinii | Bacteria | Shewanella putrefaciens | Bacteria |
| Acinetobacter guillouiae | Bacteria | Shigella boydii | Bacteria |
| Acinetobacter haemolyticus | Bacteria | Shigella dysenteriae | Bacteria |
| Acinetobacter johnsonii | Bacteria | Shigella flexneri | Bacteria |
| Acinetobacter junii | Bacteria | Shigella sonnei | Bacteria |
| Acinetobacter lwoffii | Bacteria | Sphingomonas koreensis | Bacteria |
| Acinetobacter nosocomialis | Bacteria | Staphylococcus argenteus | Bacteria |
| Acinetobacter parvus | Bacteria | Staphylococcus aureus | Bacteria |
| Acinetobacter pittii | Bacteria | Staphylococcus auricularis | Bacteria |
| Acinetobacter radioresistens | Bacteria | Staphylococcus cohnii | Bacteria |
| Acinetobacter schindleri | Bacteria | Staphylococcus condimenti | Bacteria |
| Acinetobacter seifertii | Bacteria | Staphylococcus epidermidis | Bacteria |
| Acinetobacter soli | Bacteria | Staphylococcus haemolyticus | Bacteria |
| Acinetobacter ursingii | Bacteria | Staphylococcus hominis | Bacteria |
| Actinomadura madurae | Bacteria | Staphylococcus nepalensis | Bacteria |
| Actinomyces naeslundii | Bacteria | Staphylococcus pasteuri | Bacteria |
| Actinomyces oris | Bacteria | Staphylococcus pettenkoferi | Bacteria |
| Actinomyces urogenitalis | Bacteria | Staphylococcus saprophyticus | Bacteria |
| Aeromonas caviae | Bacteria | Staphylococcus schleiferi | Bacteria |
| Aeromonas dhakensis | Bacteria | Staphylococcus warneri | Bacteria |
| Aeromonas hydrophila | Bacteria | Staphylococcus xylosus | Bacteria |
| Aeromonas jandaei | Bacteria | Stenotrophomonas maltophilia | Bacteria |
| Aeromonas media | Bacteria | Streptobacillus moniliformis | Bacteria |
| Aeromonas schubertii | Bacteria | Streptococcus acidominimus | Bacteria |
| Aeromonas sobria | Bacteria | Streptococcus agalactiae | Bacteria |
| Aeromonas veronii | Bacteria | Streptococcus alactolyticus | Bacteria |
| Aggregatibacter actinomycetemcomitans | Bacteria | Streptococcus anginosus | Bacteria |
| Alcaligenes faecalis | Bacteria | Streptococcus australis | Bacteria |
| Anaplasma phagocytophilum | Bacteria | Streptococcus canis | Bacteria |
| Arachnia propionica | Bacteria | Streptococcus constellatus | Bacteria |
| Arcanobacterium haemolyticum | Bacteria | Streptococcus cristatus | Bacteria |
| Bacillus anthracis | Bacteria | Streptococcus danieliae | Bacteria |
| Bacillus atrophaeus | Bacteria | Streptococcus dysgalactiae | Bacteria |
| Bacillus cereus | Bacteria | Streptococcus equi | Bacteria |
| Bacillus licheniformis | Bacteria | Streptococcus equinus | Bacteria |
| Bacillus pumilus | Bacteria | Streptococcus gallolyticus | Bacteria |
| Bacillus subtilis | Bacteria | Streptococcus gordonii | Bacteria |
| Bacillus thuringiensis | Bacteria | Streptococcus halichoeri | Bacteria |
| Bacteroides caccae | Bacteria | Streptococcus infantis | Bacteria |
| Bacteroides fluxus | Bacteria | Streptococcus iniae | Bacteria |
| Bacteroides fragilis | Bacteria | Streptococcus intermedius | Bacteria |
| Bacteroides pyogenes | Bacteria | Streptococcus lutetiensis | Bacteria |
| Bartonella bacilliformis | Bacteria | Streptococcus mitis | Bacteria |
| Bartonella henselae | Bacteria | Streptococcus oralis | Bacteria |
| Bartonella quintana | Bacteria | Streptococcus parasanguinis | Bacteria |
| Bordetella bronchiseptica | Bacteria | Streptococcus parasuis | Bacteria |
| Bordetella holmesii | Bacteria | Streptococcus pasteurianus | Bacteria |
| Bordetella parapertussis | Bacteria | Streptococcus pneumoniae | Bacteria |
| Bordetella pertussis | Bacteria | Streptococcus porcinus | Bacteria |
| Bordetella trematum | Bacteria | Streptococcus pseudopneumoniae | Bacteria |
| Borrelia hermsii | Bacteria | Streptococcus pseudoporcinus | Bacteria |
| Borrelia miyamotoi | Bacteria | Streptococcus pyogenes | Bacteria |
| Brevibacillus laterosporus | Bacteria | Streptococcus salivarius | Bacteria |
| Brucella abortus | Bacteria | Streptococcus sanguinis | Bacteria |
| Brucella anthropi | Bacteria | Streptococcus sobrinus | Bacteria |
| Brucella canis | Bacteria | Streptococcus suis | Bacteria |
| Brucella melitensis | Bacteria | Streptococcus uberis | Bacteria |
| Brucella suis | Bacteria | Streptococcus vestibularis | Bacteria |
| Burkholderia cenocepacia | Bacteria | Tannerella forsythia | Bacteria |
| Burkholderia cepacia | Bacteria | Treponema pallidum | Bacteria |
| Burkholderia dolosa | Bacteria | Trueperella pyogenes | Bacteria |
| Burkholderia gladioli | Bacteria | Veillonella atypica | Bacteria |
| Burkholderia glumae | Bacteria | Veillonella dispar | Bacteria |
| Burkholderia lata | Bacteria | Veillonella parvula | Bacteria |
| Burkholderia latens | Bacteria | Vibrio alginolyticus | Bacteria |
| Burkholderia mallei | Bacteria | Vibrio cholerae | Bacteria |
| Burkholderia oklahomensis | Bacteria | Vibrio furnissii | Bacteria |
| Burkholderia pseudomallei | Bacteria | Vibrio metschnikovii | Bacteria |
| Burkholderia vietnamiensis | Bacteria | Vibrio mimicus | Bacteria |
| Campylobacter coli | Bacteria | Vibrio parahaemolyticus | Bacteria |
| Campylobacter concisus | Bacteria | Vibrio vulnificus | Bacteria |
| Campylobacter curvus | Bacteria | Yersinia enterocolitica | Bacteria |
| Campylobacter fetus | Bacteria | Yersinia frederiksenii | Bacteria |
| Campylobacter hyointestinalis | Bacteria | Yersinia intermedia | Bacteria |
| Campylobacter jejuni | Bacteria | Yersinia pestis | Bacteria |
| Campylobacter lari | Bacteria | Yersinia pseudotuberculosis | Bacteria |
| Campylobacter sputorum | Bacteria | Macaca mulatta polyomavirus 1 | Virus |
| Campylobacter upsaliensis | Bacteria | Human polyomavirus 1 | Virus |
| Cedecea davisae | Bacteria | Human gammaherpesvirus 8 | Virus |
| Chlamydia abortus | Bacteria | Camelpox virus | Virus |
| Chlamydia pecorum | Bacteria | Cowpox virus | Virus |
| Chlamydia pneumoniae | Bacteria | Human parvovirus B19 | Virus |
| Chlamydia psittaci | Bacteria | Molluscum contagiosum virus | Virus |
| Chlamydia suis | Bacteria | Macacine alphaherpesvirus 1 | Virus |
| Chlamydia trachomatis | Bacteria | Macacine gammaherpesvirus 5 | Virus |
| Citrobacter amalonaticus | Bacteria | Orf virus | Virus |
| Citrobacter braakii | Bacteria | Human betaherpesvirus 5 | Virus |
| Citrobacter farmeri | Bacteria | Saimiriine gammaherpesvirus 2 | Virus |
| Citrobacter freundii | Bacteria | Mouse mammary tumor virus | Virus |
| Citrobacter koseri | Bacteria | Tanapox virus | Virus |
| Citrobacter sedlakii | Bacteria | Human mastadenovirus B | Virus |
| Citrobacter youngae | Bacteria | Rift Valley fever virus | Virus |
| Clostridioides difficile | Bacteria | Human T-lymphotropic virus 2 | Virus |
| Clostridium baratii | Bacteria | Barmah Forest virus | Virus |
| Clostridium botulinum | Bacteria | Human betaherpesvirus 7 | Virus |
| Clostridium cadaveris | Bacteria | Ebola virus | Virus |
| Clostridium disporicum | Bacteria | Taura syndrome virus | Virus |
| Clostridium novyi | Bacteria | Bunyamwera virus | Virus |
| Clostridium paraputrificum | Bacteria | Sapporo virus | Virus |
| Clostridium perfringens | Bacteria | Western equine encephalitis virus | Virus |
| Clostridium symbiosum | Bacteria | La Crosse virus | Virus |
| Clostridium tertium | Bacteria | Lymphocytic choriomeningitis mammarenavirus | Virus |
| Clostridium tetani | Bacteria | Lassa mammarenavirus | Virus |
| Comamonas aquatica | Bacteria | Simian immunodeficiency virus | Virus |
| Corynebacterium aurimucosum | Bacteria | Hendra henipavirus | Virus |
| Corynebacterium bovis | Bacteria | Eyach coltivirus | Virus |
| Corynebacterium diphtheriae | Bacteria | Colorado tick fever coltivirus | Virus |
| Corynebacterium falsenii | Bacteria | Tacaribe mammarenavirus | Virus |
| Corynebacterium jeikeium | Bacteria | Human coronavirus 229E | Virus |
| Corynebacterium macginleyi | Bacteria | Machupo mammarenavirus | Virus |
| Corynebacterium minutissimum | Bacteria | Guanarito mammarenavirus | Virus |
| Corynebacterium propinquum | Bacteria | Oropouche virus | Virus |
| Corynebacterium pseudodiphtheriticum | Bacteria | Human coronavirus NL63 | Virus |
| Corynebacterium pseudotuberculosis | Bacteria | Chikungunya virus | Virus |
| Corynebacterium striatum | Bacteria | Crimean-Congo hemorrhagic fever orthonairovirus | Virus |
| Corynebacterium ulcerans | Bacteria | Sin Nombre orthohantavirus | Virus |
| Corynebacterium xerosis | Bacteria | Measles morbillivirus | Virus |
| Coxiella burnetii | Bacteria | Omsk hemorrhagic fever virus | Virus |
| Cronobacter dublinensis | Bacteria | Getah virus | Virus |
| Cronobacter malonaticus | Bacteria | Brazilian mammarenavirus | Virus |
| Cronobacter sakazakii | Bacteria | Alphacoronavirus 1 | Virus |
| Cutibacterium acnes | Bacteria | Human immunodeficiency virus 2 | Virus |
| Dermatophilus congolensis | Bacteria | Respiratory syncytial virus | Virus |
| Edwardsiella tarda | Bacteria | Argentinian mammarenavirus | Virus |
| Ehrlichia chaffeensis | Bacteria | Hepatitis delta virus | Virus |
| Eikenella corrodens | Bacteria | Mopeia mammarenavirus | Virus |
| Elizabethkingia meningoseptica | Bacteria | Mumps orthorubulavirus | Virus |
| Enterobacter asburiae | Bacteria | Monkeypox virus | Virus |
| Enterobacter bugandensis | Bacteria | Marburg marburgvirus | Virus |
| Enterobacter cancerogenus | Bacteria | Yellow fever virus | Virus |
| Enterobacter chengduensis | Bacteria | Human alphaherpesvirus 3 | Virus |
| Enterobacter chuandaensis | Bacteria | Human alphaherpesvirus 2 | Virus |
| Enterobacter cloacae | Bacteria | Human coronavirus HKU1 | Virus |
| Enterobacter hormaechei | Bacteria | Rabies lyssavirus | Virus |
| Enterobacter huaxiensis | Bacteria | Variola virus | Virus |
| Enterobacter kobei | Bacteria | Human alphaherpesvirus 1 | Virus |
| Enterobacter ludwigii | Bacteria | Vaccinia virus | Virus |
| Enterobacter mori | Bacteria | Semliki Forest virus | Virus |
| Enterobacter quasiroggenkampii | Bacteria | Powassan virus | Virus |
| Enterobacter roggenkampii | Bacteria | Hepatovirus A | Virus |
| Enterobacter sichuanensis | Bacteria | Sindbis virus | Virus |
| Enterococcus avium | Bacteria | Langat virus | Virus |
| Enterococcus casseliflavus | Bacteria | West Nile virus | Virus |
| Enterococcus durans | Bacteria | Orthohepevirus A | Virus |
| Enterococcus faecalis | Bacteria | Enterovirus C | Virus |
| Enterococcus faecium | Bacteria | Rhinovirus B | Virus |
| Enterococcus gallinarum | Bacteria | Hepatitis B virus | Virus |
| Enterococcus mundtii | Bacteria | Hepacivirus C | Virus |
| Erysipelatoclostridium ramosum | Bacteria | Coxsackievirus A16 | Virus |
| Erysipelothrix rhusiopathiae | Bacteria | Venezuelan equine encephalitis virus | Virus |
| Escherichia marmotae | Bacteria | Dengue virus | Virus |
| Francisella tularensis | Bacteria | Japanese encephalitis virus | Virus |
| Fusobacterium gonidiaformans | Bacteria | Ross River virus | Virus |
| Fusobacterium necrophorum | Bacteria | Betacoronavirus 1 | Virus |
| Fusobacterium nucleatum | Bacteria | Eastern equine encephalitis virus | Virus |
| Fusobacterium periodonticum | Bacteria | Avian orthoavulavirus 1 | Virus |
| Fusobacterium ulcerans | Bacteria | Gammacoronavirus | Virus |
| Fusobacterium varium | Bacteria | Onyong-nyong virus | Virus |
| Gardnerella vaginalis | Bacteria | Rubivirus rubellae | Virus |
| Gemella haemolysans | Bacteria | Tick-borne encephalitis virus | Virus |
| Gemella sanguinis | Bacteria | Louping ill virus | Virus |
| Haemophilus aegyptius | Bacteria | Foot-and-mouth disease virus | Virus |
| Haemophilus ducreyi | Bacteria | Kyasanur Forest disease virus | Virus |
| Haemophilus haemolyticus | Bacteria | Mayaro virus | Virus |
| Haemophilus influenzae | Bacteria | Murray Valley encephalitis virus | Virus |
| Haemophilus parahaemolyticus | Bacteria | Human T-cell leukemia virus type I | Virus |
| Haemophilus paraphrohaemolyticus | Bacteria | Nipah henipavirus | Virus |
| Hafnia alvei | Bacteria | JC polyomavirus | Virus |
| Helicobacter bilis | Bacteria | Mus musculus polyomavirus 1 | Virus |
| Helicobacter cinaedi | Bacteria | Norwalk virus | Virus |
| Helicobacter pullorum | Bacteria | Rotavirus H | Virus |
| Helicobacter pylori | Bacteria | Human immunodeficiency virus 1 | Virus |
| Hungatella hathewayi | Bacteria | Severe acute respiratory syndrome-related coronavirus | Virus |
| Kingella kingae | Bacteria | Human metapneumovirus | Virus |
| Klebsiella aerogenes | Bacteria | Saint Louis encephalitis virus | Virus |
| Klebsiella michiganensis | Bacteria | Sepik virus | Virus |
| Klebsiella oxytoca | Bacteria | Human gammaherpesvirus 4 | Virus |
| Klebsiella pneumoniae | Bacteria | Flexal mammarenavirus | Virus |
| Klebsiella variicola | Bacteria | Astrovirus MLB1 | Virus |
| Lacticaseibacillus rhamnosus | Bacteria | Human T-lymphotropic virus 4 | Virus |
| Lactiplantibacillus plantarum | Bacteria | Zika virus | Virus |
| Lactobacillus acidophilus | Bacteria | Cercopithecine betaherpesvirus 5 | Virus |
| Lactobacillus gasseri | Bacteria | Astrovirus VA1 | Virus |
| Lactobacillus jensenii | Bacteria | Rotavirus D | Virus |
| Lactobacillus paragasseri | Bacteria | Bebaru virus | Virus |
| Lactococcus garvieae | Bacteria | Astrovirus MLB2 | Virus |
| Lactococcus lactis | Bacteria | Ndumu virus | Virus |
| Legionella jordanis | Bacteria | Dabie bandavirus | Virus |
| Legionella longbeachae | Bacteria | Simbu orthobunyavirus | Virus |
| Legionella pneumophila | Bacteria | Astrovirus VA4 | Virus |
| Leptospira interrogans | Bacteria | Aino orthobunyavirus | Virus |
| Leptospira meyeri | Bacteria | Mink calicivirus | Virus |
| Limosilactobacillus fermentum | Bacteria | Middle East respiratory syndrome-related coronavirus | Virus |
| Listeria grayi | Bacteria | Astrovirus VA3 | Virus |
| Listeria innocua | Bacteria | Hepacivirus E | Virus |
| Listeria ivanovii | Bacteria | Human parainfluenza virus 4a | Virus |
| Listeria monocytogenes | Bacteria | Razdan bandavirus | Virus |
| Mannheimia haemolytica | Bacteria | Middelburg virus | Virus |
| Methylorubrum extorquens | Bacteria | Heartland bandavirus | Virus |
| Microbacterium hominis | Bacteria | unclassified Peribunyaviridae | Virus |
| Micrococcus luteus | Bacteria | Human papillomavirus | Virus |
| Moraxella catarrhalis | Bacteria | Rotavirus G | Virus |
| Moraxella nonliquefaciens | Bacteria | Arurhavirus inhangapi | Virus |
| Morganella morganii | Bacteria | Flanders hapavirus | Virus |
| Mycobacterium asiaticum | Bacteria | Saumarez Reef virus | Virus |
| Mycobacterium avium | Bacteria | Wyeomyia orthobunyavirus | Virus |
| Mycobacterium goodii | Bacteria | Dhori thogotovirus | Virus |
| Mycobacterium kansasii | Bacteria | Nairobi sheep disease orthonairovirus | Virus |
| Mycobacterium malmoense | Bacteria | Caraparu orthobunyavirus | Virus |
| Mycobacterium tuberculosis | Bacteria | Nyando orthobunyavirus | Virus |
| Mycobacterium ulcerans | Bacteria | Guaroa orthobunyavirus | Virus |
| Mycobacterium xenopi | Bacteria | Bwamba orthobunyavirus | Virus |
| Mycobacteroides chelonae | Bacteria | Hart Park virus | Virus |
| Mycolicibacterium fortuitum | Bacteria | Curiovirus rochambeau | Virus |
| Mycolicibacterium smegmatis | Bacteria | Tacaiuma orthobunyavirus | Virus |
| Mycoplasma capricolum | Bacteria | Turlock orthobunyavirus | Virus |
| Mycoplasma pneumoniae | Bacteria | Everglades virus | Virus |
| Neisseria cinerea | Bacteria | Mucambo virus | Virus |
| Neisseria flavescens | Bacteria | Hazara virus | Virus |
| Neisseria gonorrhoeae | Bacteria | Sagiyama virus | Virus |
| Neisseria meningitidis | Bacteria | Human immunodeficiency virus | Virus |
| Neisseria mucosa | Bacteria | California encephalitis virus | Virus |
| Neisseria subflava | Bacteria | Rocio virus | Virus |
| Nocardia abscessus | Bacteria | Kunjin virus | Virus |
| Nocardia asteroides | Bacteria | Tensaw virus | Virus |
| Nocardia brasiliensis | Bacteria | Jamestown Canyon orthobunyavirus | Virus |
| Nocardia farcinica | Bacteria | Anhembi orthobunyavirus | Virus |
| Nocardia nova | Bacteria | Macaua orthobunyavirus | Virus |
| Nocardia otitidiscaviarum | Bacteria | Ilesha orthobunyavirus | Virus |
| Paeniclostridium sordellii | Bacteria | Tataguine orthobunyavirus | Virus |
| Pantoea agglomerans | Bacteria | Enseada orthobunyavirus | Virus |
| Parvimonas micra | Bacteria | Fort Sherman orthobunyavirus | Virus |
| Pasteurella multocida | Bacteria | Cache Valley orthobunyavirus | Virus |
| Peptostreptococcus anaerobius | Bacteria | Keystone orthobunyavirus | Virus |
| Phocaeicola dorei | Bacteria | Tahyna orthobunyavirus | Virus |
| Plesiomonas shigelloides | Bacteria | Melao orthobunyavirus | Virus |
| Porphyromonas gingivalis | Bacteria | Maguari orthobunyavirus | Virus |
| Prevotella amnii | Bacteria | Shuni orthobunyavirus | Virus |
| Prevotella bivia | Bacteria | unidentified adenovirus | Virus |
| Prevotella buccae | Bacteria | Rabbitpox virus | Virus |
| Prevotella buccalis | Bacteria | Guinea pig adenovirus | Virus |
| Prevotella copri | Bacteria | Echovirus E9 | Virus |
| Prevotella corporis | Bacteria | Enterovirus A71 | Virus |
| Prevotella denticola | Bacteria | Human rhinovirus AMS323 | Virus |
| Prevotella disiens | Bacteria | Coxsackievirus A9 | Virus |
| Prevotella histicola | Bacteria | Echovirus E6 | Virus |
| Prevotella intermedia | Bacteria | Germiston virus | Virus |
| Prevotella melaninogenica | Bacteria | Oropouche orthobunyavirus | Virus |
| Prevotella nanceiensis | Bacteria | Bunyamwera orthobunyavirus | Virus |
| Prevotella nigrescens | Bacteria | Apeu orthobunyavirus | Virus |
| Prevotella oris | Bacteria | California encephalitis orthobunyavirus | Virus |
| Prevotella pallens | Bacteria | unclassified Orthopoxvirus | Virus |
| Prevotella ruminicola | Bacteria | Tai Forest coltivirus | Virus |
| Prevotella salivae | Bacteria | Itaituba phlebovirus | Virus |
| Prevotella timonensis | Bacteria | Gordil phlebovirus | Virus |
| Proteus mirabilis | Bacteria | Sunrhavirus garba | Virus |
| Proteus penneri | Bacteria | Issyk-kul orthonairovirus | Virus |
| Proteus vulgaris | Bacteria | Tamdy orthonairovirus | Virus |
| Providencia alcalifaciens | Bacteria | Mapputta orthobunyavirus | Virus |
| Providencia rettgeri | Bacteria | Buffalo Creek orthobunyavirus | Virus |
| Providencia stuartii | Bacteria | Astrovirus VA2 | Virus |
| Pseudomonas aeruginosa | Bacteria | Astrovirus SG | Virus |
| Pseudomonas fulva | Bacteria | Human rhinovirus sp. | Virus |
| Pseudomonas koreensis | Bacteria | Coxsackievirus | Virus |
| Pseudomonas monteilii | Bacteria | Human enterovirus | Virus |
| Pseudomonas oryzihabitans | Bacteria | Human rhinovirus strain Hanks | Virus |
| Pseudomonas putida | Bacteria | Echovirus | Virus |
| Ralstonia pickettii | Bacteria | Human echovirus AMS573 | Virus |
| Rhodococcus equi | Bacteria | Human echovirus AMS721 | Virus |
| Rickettsia conorii | Bacteria | Sapovirus Sapozj-9 | Virus |
| Rickettsia japonica | Bacteria | Influenza A Virus | Virus |
| Rodentibacter pneumotropicus | Bacteria | Influenza B Virus | Virus |
| Rothia dentocariosa | Bacteria | Influenza C Virus | Virus |

**S5. Pathogen Sequence Typing**

The tool accepts assembly data in FASTA format and employs Snippy v.4.6.0 and iVar 1.3.1 for SNP calling on pathogenic bacteria and viruses, utilizing reference sequences from the Reference Database. Subsequently, a Core SNP matrix is constructed using Gubbins v.3.3.0 [18] to eliminate SNPs in recombination regions. Based on these results, the tool also allows users to upload more than three sequences and associated metadata for phylogenetic tree reconstruction.

**S6. Genomic Annotation**

Genomic Annotation starts with genomic component analysis using PILER-CR v.1.06 [19] for CRISPR array recognition, followed by repeated structure detection using TRF v.4.07b [20]. Non-coding RNA prediction is then carried out with tRNAscanSE v.1.4 [21] and RNA mmer v.1.2. Finally, gene prediction is performed using Prodigal v.2.6.3 [22]. The predicted genes can be selected for annotation against 11 commonly used functional databases, including KEGG [23], COG [24], NCBI-nr [25], CARD [26], CAZy [27], PHI [28], SwissProt [29], VFDB [30], Pfam [31], MetaCyc [32], and antiSMASH [33].

**S7. MGE, ARG and VF Detection**

This analysis tool includes seven commonly used MGE annotation programs: ISEScan [34], DANMEL [35], MobileElementFinder [36], BacAnt [37], IntegreonFinder [38], Platon [39], and PlasmidFinder [40]. Furthermore, ARGs are predicted using Dimond based on CARD database. And VFs were predicted by Dimond based on the VFDB database.

And the criteria for the prediction of transferable ARGs and VFs [41] are as follw: 1) If an ARG or VF contains the same IS on both sides within 10 kb upstream and downstream of the coding sequence, it is considered to have transferability. 2) If the position of an ARG or VF is within the sequence range of an ICE, IN, plasmid, phage, or Tn, it is considered to have the potential for horizontal transfer. This tool, for the first time, combines MGE annotation with the detection of horizontally transferable ARGs and VFs detection, quickly identifying MGEs, ARGs, and VFs with transfer risk in pathogenic bacteria.

**References**

[1] H. Li, R. Durbin, Fast and accurate short read alignment with Burrows-Wheeler transform, Bioinformatics. 25 (2009) 1754-1760, <https://doi.org/10.1093/bioinformatics/btp324>.

[2] T. N. Nguyen, C. Gonzalez, Minimap: An interactive dynamic decision making game for search and rescue missions, Behav. Res. Methods. (2023) Epub ahead of print. https://doi.org/10.3758/s13428-023-02149-7.

[3] S. Castellano, F. Cestari, G. Faglioni, E. Tenedini, M, Marino, L. Artuso, R. Manfredini, M. Luppi, T. Trenti, E. Tagliafico, iVar, an Interpretation-Oriented Tool to Manage the Update and Revision of Variant Annotation and Classification, Genes (Basel). 12 (2021) 384, https://doi.org/10.3390/genes12030384.

[4] A. Gurevich, V. Saveliev, N. Vyahhi, G. Tesler, QUAST: quality assessment tool for genome assemblies, Bioinformatics. 29 (2013) 1072-1075, https://doi.org/10.1093/bioinformatics/btt086.

[5] P. Stothard, D. S. Wishart, Circular genome visualization and exploration using CGView, Bioinformatics. 21 (2005) 537-539, https://doi.org/10.1093/bioinformatics/bti054.

[6] Y. Xie, G. Wu, J. Tang, R. Luo, J. Patterson, S. Liu, W. Huang, G. He, S. Gu, S. Li, et al., SOAPdenovo-Trans: de novo transcriptome assembly with short RNA-Seq reads, Bioinformatics. 30 (2014) 1660-1666, https://doi.org/10.1093/bioinformatics/btu077.

[7] A. Bankevich, S. Nurk, D. Antipov, A. A. Gurevich, M. Dvorki, A. S. Kulikov, V. M. Lesin, S. I. Nikolenko, S. Pham, A. D. Prjibelski, et al., SPAdes: a new genome assembly algorithm and its applications to single-cell sequencing, J. Comput. Biol. 19 (2012) 455-477, https://doi.org/10.1089/cmb.2012.0021.

[8] R. Kajitani, Y. Ogura, Y. Gotoh, T. Hayashi, T. Itoh, Platanus_B: an accurate de novo assembler for bacterial genomes using an iterative error-removal process, DNA Res. 27 (2020) dsaa014, https://doi.org/10.1093/dnares/dsaa014.

[9] D. R. Zerbino, E. Birney, Velvet: algorithms for de novo short read assembly using de Bruijn graphs, Genome. Res. 18 (2008) 821-829, http://doi.org/10.1101/gr.074492.107.

[10] Y. Peng, H. C. Leung, S. M. Yiu, F. Y. Chin, IDBA-UD: a de novo assembler for single-cell and metagenomic sequencing data with highly uneven depth, Bioinformatics. 28 (2012) 1420-1428, https://doi.org/10.1093/bioinformatics/bts174.

[11] D. H. Parks, M. Imelfort, C. T. Skennerton, P. Hugenholtz, G. W. Tyson, CheckM: assessing the quality of microbial genomes recovered from isolates, single cells, and metagenomes, Genome. Res. 25 (2015) 1043-1055, https://doi.org/10.1101/gr.186072.114.

[12] G. M. Boratyn, J. Thierry-Mieg, D. Thierry-Mieg, B. Busby, T. L. Madden, Magic-BLAST, an accurate RNA-seq aligner for long and short reads, BMC. Bioinformatics. 20 (2019) 405, https://doi.org/10.1186/s12859-019-2996-x.

[13] B. D. Ondov, T. J. Treangen, P. Melsted, A. B. Mallonee, N. H. Bergman, S. Koren, A. M. Phillippy, Mash: fast genome and metagenome distance estimation using MinHash, Genome. Biol. 17 (2016) 132, https://doi.org/10.1186/s13059-016-0997-x.

[14] I. Lee, Y. K. Ouk, S. C. Park, J. Chun, OrthoANI: An improved algorithm and software for calculating average nucleotide identity, Int. J. Syst. Evol. Microbiol. 66 (2016) 1100-1103, https://doi.org/10.1099/ijsem.0.000760.

[15] R. C. Edgar, Search and clustering orders of magnitude faster than BLAST, Bioinformatics. 26 (2010) 2460-2461, https://doi.org/10.1093/bioinformatics/btq461.

[16] J. E. Hernández-Salmerón, G. Moreno-Hagelsieb, FastANI, Mash and Dashing equally differentiate between Klebsiella species, PeerJ. 10 (2022) e13784, https://doi.org/10.7717/peerj.

[17] D. E. Wood, J. Lu, B. Langmead, Improved metagenomic analysis with Kraken 2, Genome. Biol. 20 (2019) 257, https://doi.org/10.1186/s13059-019-1891-0.

[18] N. J. Croucher, A. J. Page, T. R. Connor, A. J. Delaney, J. A. Keane, S. D. Bentley, J. Parkhill, S. R. Harris, Rapid phylogenetic analysis of large samples of recombinant bacterial whole genome sequences using Gubbins, Nucleic. Acids. Res. 43 (2015) e15, https://doi.org/10.1093/nar/gku1196.

[19] R. C. Edgar, PILER-CR: fast and accurate identification of CRISPR repeats, BMC. Bioinformatics. 8 (2007) 18, https://doi.org/10.1186/1471-2105-8-18.

[20] T. W. Gant, U. G. Sauer, S. D. Zhang, B. N. Chorley, J. Hackermüller, S. Perdichizzi, K. E. Tollefsen, B. van Ravenzwaay, C. Yauk, W. Tong, et al., A generic Transcriptomics Reporting Framework (TRF) for 'omics data processing and analysis, Regul. Toxicol. Pharmacol. 91 (2017) S36-S45, https://doi.org/10.1016/j.yrtph.2017.11.001.

[21] P. P. Chan, B. Y. Lin, A. J. Mak, T. M. Lowe, tRNAscan-SE 2.0: improved detection and functional classification of transfer RNA genes, Nucleic. Acids. Res. 49 (2021) 9077-9096, https://doi.org/10.1093/nar/gkab688.

[22] D. Hyatt, G. L. Chen, P. F. Locascio, M. L. Land, F. W. Larimer, L. J. Hauser, Prodigal: prokaryotic gene recognition and translation initiation site identification, BMC. Bioinformatics. 11 (2010) 119, https://doi.org/10.1186/1471-2105-11-119.

[23] M. Kanehisa, S. Goto, KEGG: kyoto encyclopedia of genes and genomes, Nucleic. Acids. Res, 28 (2000) 27-30, https://doi.org/10.1093/nar/28.1.27.

[24] M. Y. Galperin, K. S. Makarova, R. Vera Alvarez, D. Landsman, E. V. Koonin, COG database update: focus on microbial diversity, model organisms, and widespread pathogens, Nucleic. Acids. Res. 49 (2021) D274-D281, https://doi.org/10.1093/nar/gkaa1018.

[25] D. A. Benson, I. Karsch-Mizrachi, D. J. Lipman, J. Ostell, D. L. Wheeler, GenBank, Nucleic. Acids. Res. 33 (2005) D34-D38, https://doi.org/10.1093/nar/gki063.

[26] B. P. Alcock, A. R. Raphenya, T. T. Y. Lau, K. K. Tsang, M. Bouchard, A. Edalatmand, W. Huynh, A. V. Nguyen, A. A. Cheng, S. Liu, et al., CARD 2020: antibiotic resistome surveillance with the comprehensive antibiotic resistance database, Nucleic. Acids. Res. 48 (2020) D517-D525, https://doi.org/10.1093/nar/gkz935.

[27] V. Lombard, E. Drula, P. M. Coutinho, B. Henrissat, The carbohydrate-active enzymes database (CAZy) in 2013, Nucleic. Acids. Res. 42 (2013) D490-D495, https://doi.org/10.1093/nar/gkt1178.

[28] M. Urban, A. Cuzick, J. Seager, V. Wood, K. Rutherford, S. Y. Venkatesh, N. De Silva, M. C. Martinez, H. Pedro, A. D. Yates, et al., PHI-base: the pathogen-host interactions database, Nucleic. Acids. Res. 48 (2020) D613-D620, https://doi.org/10.1093/nar/gkz904.

[29] L. E. McMillan, A. C. Martin, Automatically extracting functionally equivalent proteins from SwissProt, BMC. Bioinformatics. 9 (2008) 418, https://doi.org/10.1186/1471-2105-9-418.

[30] B. Liu, D. Zheng, S. Zhou, L. Chen, J. Yang, VFDB 2022: a general classification scheme for bacterial virulence factors, Nucleic. Acids. Res. 50 (2022) D912-D917, https://doi.org/10.1093/nar/gkab1107.

[31] J. Mistry, S. Chuguransky, L. Williams, M. Qureshi, G. A. Salazar, E. L. L. Sonnhammer, S. C. E. Tosatto, L. Paladin, S. Raj, L. J. Richardson, et al., Pfam: The protein families database in 2021, Nucleic. Acids. Res. 49 (2021) D412-D419, https://doi.org/10.1093/nar/gkaa913.

[32] P. D. Karp, M. Riley, S. M. Paley, Pellegrini-Toole A, The MetaCyc Database, Nucleic. Acids. Res. 30 (2002) 59-61, https://doi.org/10.1093/nar/30.1.59.

[33] K. Blin, S. Shaw, A. M. Kloosterman, Z. Charlop-Powers, G. P. van Wezel, M. H. Medema, T. Weber, antiSMASH 6.0: improving cluster detection and comparison capabilities, Nucleic. Acids. Res. 49 (2021) W29-W35, https://doi.org/10.1093/nar/gkab335.

[34] Z. Xie, H. Tang ISEScan: automated identification of insertion sequence elements in prokaryotic genomes, Bioinformatics. 33 (2017) 3340-3347, https://doi.org/10.1093/bioinformatics/btx433.

[35] P. Wang, X. Jiang, K. Mu, Y. Jing, Z. Yin, Y. J. Cui, C. Li, X. H. Luo, F. Z. Chen, T. Yu, et al., DANMEL: A manually curated reference database for analyzing mobile genetic elements associated with bacterial drug resistance, mLife. 1 (2022) 460-464, https://doi.org/10.1002/mlf2.12046.

[36] M. H. K. Johansson, V. Bortolaia, S. Tansirichaiya, F. M. Aarestrup, A. P. Roberts, T. N. Petersen, Detection of mobile genetic elements associated with antibiotic resistance in Salmonella enterica using a newly developed web tool: MobileElementFinder, J. Antimicrob. Chemother. 76 (2021) 101-109, https://doi.org/10.1093/jac/dkaa390.

[37] X. Hua, Q. Liang, M. Deng, J. He, M. Wang, W. Hong, J. Wu, B. Lu, S. Leptihn, Y. Yu, et al., BacAnt: A Combination Annotation Server for Bacterial DNA Sequences to Identify Antibiotic Resistance Genes, Integrons, and Transposable Elements, Front. Microbiol. 12 (2021) 649969, https://doi.org/10.3389/fmicb.2021.649969.

[38] B. Néron, E. Littner, M. Haudiquet, A. Perrin, J. Cury, E. P. C. Rocha, IntegronFinder 2.0: Identification and Analysis of Integrons across Bacteria, with a Focus on Antibiotic Resistance in Klebsiella, Microorganisms. 10 (2022) 700, https://doi.org/10.3390/microorganisms10040700.

[39] O. Schwengers, P. Barth, L. Falgenhauer, T. Hain, T. Chakraborty, A. Goesmann, Platon: identification and characterization of bacterial plasmid contigs in short-read draft assemblies exploiting protein sequence-based replicon distribution scores, Microb. Genom. 6 (2020) mgen000398, https://doi.org/10.1099/mgen.0.000398.

[40] A. Carattoli, H. Hasman, PlasmidFinder and In Silico pMLST: Identification and Typing of Plasmid Replicons in Whole-Genome Sequencing (WGS), Methods. Mol. Biol. 2075 (2020) 285-294, https://doi.org/10.1007/978-1-4939-9877-7_20.

[41] S. R. Partridge, S. M. Kwong, N. Firth, S. O. Jensen, Mobile Genetic Elements Associated with Antimicrobial Resistance, Clin. Microbiol. Rev. 31 (2018) e00088-e000817, https://doi.org/10.1128/CMR.00088-17.
